# Timing of progression from *Chlamydia trachomatis* infection to pelvic inflammatory disease: a mathematical modelling study

**DOI:** 10.1186/1471-2334-12-187

**Published:** 2012-08-11

**Authors:** Sereina A Herzog, Christian L Althaus, Janneke CM Heijne, Pippa Oakeshott, Sally Kerry, Phillip Hay, Nicola Low

**Affiliations:** 1 Institute of Social and Preventive Medicine (ISPM), University of Bern, Bern, CH-3012, Switzerland; 2 Division of Population Health Sciences and Education, St George’s, University of London, London, SW17 0RE, UK; 3 Department of Genitourinary Medicine, St George’s Hospital, London, SW17 0QT, UK; 4 Centre for Primary Care and Public Health, Blizard Institute, Queen Mary University of London, London, E12AB, UK

**Keywords:** Chlamydia infection, Pelvic inflammatory disease, Mathematical model, Compartmental model, Randomised controlled trials

## Abstract

**Background:**

Pelvic inflammatory disease (PID) results from the ascending spread of microorganisms from the vagina and endocervix to the upper genital tract. PID can lead to infertility, ectopic pregnancy and chronic pelvic pain. The timing of development of PID after the sexually transmitted bacterial infection *Chlamydia trachomatis* (chlamydia) might affect the impact of screening interventions, but is currently unknown. This study investigates three hypothetical processes for the timing of progression: at the start, at the end, or throughout the duration of chlamydia infection.

**Methods:**

We develop a compartmental model that describes the trial structure of a published randomised controlled trial (RCT) and allows each of the three processes to be examined using the same model structure. The RCT estimated the effect of a single chlamydia screening test on the cumulative incidence of PID up to one year later. The fraction of chlamydia infected women who progress to PID is obtained for each hypothetical process by the maximum likelihood method using the results of the RCT.

**Results:**

The predicted cumulative incidence of PID cases from all causes after one year depends on the fraction of chlamydia infected women that progresses to PID and on the type of progression. Progression at a constant rate from a chlamydia infection to PID or at the end of the infection was compatible with the findings of the RCT. The corresponding estimated fraction of chlamydia infected women that develops PID is 10% (95% confidence interval 7-13%) in both processes.

**Conclusions:**

The findings of this study suggest that clinical PID can occur throughout the course of a chlamydia infection, which will leave a window of opportunity for screening to prevent PID.

## Background

Pelvic inflammatory disease (PID) is a clinical syndrome resulting from the ascending spread of microorganisms from the vagina and endocervix to the endometrium, fallopian tubes, and/or contiguous structures [1]. Damage to the fallopian tubes following PID is a predisposing factor for ectopic pregnancy and infertility [2]. *Chlamydia trachomatis* (chlamydia) has been found in approximately 30% of all PID cases [2,3] and is the most common bacterial sexually transmitted infection in many developed countries [4]. Chlamydia infection is usually asymptomatic in women, but can be treated with antibiotics when diagnosed [5]. The estimated mean duration of untreated asymptomatic infection is more than one year in women [6,7].

Early detection and treatment of chlamydia through screening has been proposed as a strategy to prevent PID and subsequent reproductive tract morbidity in sexually active young women [8]. Three randomised controlled trials have investigated the efficacy of a single chlamydia screening test on the incidence of clinically diagnosed PID with a follow-up period of one year in young women [9-11]. Uptake of screening ranged from 64 to 100% and all three trials found a reduction in the incidence of PID from any cause in the intervention group compared to the control group.

It is important to understand when in the course of infection PID occurs and when screening and treatment should take place to maximise the potential of chlamydia screening to prevent PID, but this is currently unknown. The natural history of untreated chlamydia in humans cannot be directly observed for ethical and logistical reasons and randomised controlled trials do not provide this information because the time from the start of infection is unknown. It has been suggested that treatment is needed soon after infection, based on observations from an animal model [12]. Pal et al. isolated the *C. trachomatis* mouse pneumonitis biovar from the upper genital tract 24 hours after vaginal inoculation in mice [12].

Mathematical modelling studies are a valuable tool for investigating hypothetical processes of chlamydia transmission and ascending infection. Amongst the few mathematical modelling studies with explicit descriptions of progression from chlamydia infection to PID, it has been proposed that PID develops in the first half of a chlamydia infection, in the second half, or can occur at any time during a chlamydia infection [13]. The objectives of this study were: to investigate how differences in the timing of progression from chlamydia infection to PID affect the outcome of a chlamydia screening intervention; and to estimate the fraction of chlamydia infections that progresses to PID, using a mathematical model to simulate the results of a published randomised controlled trial.

## Methods

### Data

We used data from the Prevention Of Pelvic Infection (POPI) randomised controlled trial of chlamydia screening, which provides information about *C. trachomatis* infection status at baseline in both the intervention and the control groups and about incident clinically diagnosed PID up to one year later [11,14]. In brief, the study enrolled about 2500 sexually active women aged 16 to 24 years from colleges and universities in London. All women provided self-collected vaginal swabs at enrolment and were randomised to immediate testing for chlamydia infection and treatment if positive (intervention group), or the collected swabs were stored and tested after one year (control group). The prevalence of chlamydia infection was 5.4% (68/1254) in the intervention group and 5.9% (75/1265) in the control group, i.e. overall 5.7% (143/2519). About 22.2% (527/2377) of the women in both groups reported being tested independently for chlamydia during the follow-up period. The incidence of clinically diagnosed PID (by self-report, mostly backed up by examination of medical records) after one year was 1.3% (15/1191, 95% CI 0.7 to 2.1%) in the intervention group and 1.9% (23/1186, 95% CI 1.2 to 2.9%) in the control group. The incidence rates of PID in women with chlamydia infection at baseline were 1.6% (1/63) in the intervention group and 9.5% (7/74) in the control group. Amongst women in the control group who developed PID, 30.0% (7/23) were chlamydia positive at baseline.

### Model

**Figure 1:**
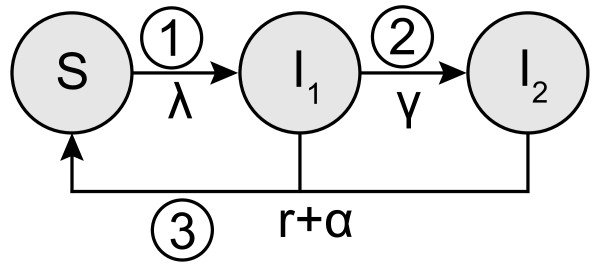
**Schematic overview of the model framework.** The model has a susceptible-infected-susceptible (SIS) framework and allows three hypothetical processes for the timing of progression from chlamydia to PID to be investigated. A woman can become infected at rate *λ* (force of infection), can clear her infection naturally (rate *r*), or can be effectively screened and treated (rate *α*). Numbers indicate when during the chlamydia infection progression to PID could happen: 1) immediate progression, 2) constant progression, and 3) progression at the end of infection. For all three types of progression a certain fraction *f* of chlamydia-infected women will develop PID. For the constant progression model a woman moves from being infected without PID (I_1_) to being infected with PID (I_2_) at rate *γ*, which is set to $\gamma =\frac{f}{1-f}r$. For immediate progression and the progression at the end of infection we set γ=0 and $\text{I}={\text{I}}_{1}+{\text{I}}_{2}$.

We developed a compartmental model that describes the trial structure using a Susceptible-Infected-Susceptible (SIS) framework (Figure 1). We assume a closed population of susceptible (S) women who can become infected (I) at constant rate *λ*, i.e., the force of infection, and clear the infection naturally at rate *r.* The infection is separated into two stages so that we can distinguish between infected women without PID (I_1_) and with PID (I_2_). The transition from the first to the second stage happens at the progression rate *γ* and allows us to investigate different possibilities for the timing of progression in the same model. During follow-up a woman can receive a test and is successfully treated at rate *α*, which incorporates the percentage of women ( *c*) who reported being tested for chlamydia during the follow-up period and the proportion with treatment failure ( *δ*). This results in the following system of ordinary differential equations:

$$\begin{array}{l} \frac{dS\left(t\right)}{dt}=-\lambda S(t)+(r+\alpha )(I_{1}(t)+I_{2}(t))\\ \frac{dI_{1}\left(t\right)}{dt}=\lambda S\left(t\right)-\left(r+\alpha +\gamma \right)I_{1}\left(t\right)\\ \frac{dI_{2}\left(t\right)}{dt}=\gamma I_{1}\left(t\right)-\left(r+\alpha \right)I_{2}\left(t\right) \end{array}$$

The force of infection *λ* is assumed to be constant over time because the study population is small compared to the population in which the study took place so changes in prevalence within the study population are unlikely to affect the overall chlamydia prevalence. The force of infection *λ* is calibrated so that the steady state prevalence in the model is equal to the prevalence *p* at baseline in the absence of the trial ( *α* = 0). We assume the infection duration to be exponentially distributed [6] with a mean duration of *1/r*. This takes into account the fact that that some women clear the infection rapidly whereas others can remain infected for substantially longer time periods [7].

At model initiation we simulate the conditions in the two arms in the trial. In the control group, a percentage of women is infected, reflecting the observed baseline prevalence. In the intervention group all women have received treatment but a small percentage remains infected owing to treatment failure (see Additional file 1 for more details).

### Types of progression

We explored three hypothetical processes for the timing of progression from endocervical *C. trachomatis* infection to PID (Figure 1). For each type of progression it is assumed that, of all women infected, a certain fraction *f* will develop PID in the absence of an intervention. The first possibility is that PID develops at the start of a chlamydia infection (immediate progression); the incidence of PID depends on the force of infection and the number of susceptible women ( *fλ*S), so we set *γ* = 0 and I = I_1_ + I_2_. The second possibility is that PID can develop at a constant rate throughout the course of a chlamydia infection (constant progression); the incidence of PID depends on the progression rate *γ* and the number of women in the infected compartment without PID ( *γ*I_1_). The progression rate is defined as $\gamma =\frac{f}{1-f}r$ and the mean duration of the infection is consistent with the other two types of progression. Finally, progression to PID could happen at the end of a chlamydia infection just before natural clearance (progression at the end). In this situation, PID incidence depends on the clearance rate and the number of infected women (*fr*I), where *γ* = 0 and I = I_1_ + I_2_. In the absence of the trial (*α* = 0), the incidence rates of PID are the same for all three types of progression. The cumulative incidence of PID cases caused by *C. trachomatis* is tracked for both groups and is set to zero at model initiation (Additional file 1).

### Proportion of PID cases caused by *C. trachomatis*

The observed numbers of PID cases in the intervention and control group are presumed to be a mixture of PID cases caused by *C. trachomatis* and by other microorganisms. We assume that a certain proportion *x* of PID cases in the control group is caused by chlamydia and that the amount caused by other microorganisms is the same in both groups. In the simulated trial it is assumed that the intervention only reduces the incidence of chlamydial PID.

The model estimates the cumulative incidence of chlamydial PID for the intervention group (*g*_*I*_) and for the control group (*g*_*C*_). We get the overall cumulative incidence of PID cases in the intervention group (*e*_*I*_) and in the control group (*e*_*C*_) by using the proportion of PID cases caused by chlamydia (*x*), as follows:

$$\begin{array}{l} e_{C}=\frac{g_{C}}{x}\\ e_{I}=g_{I}+(e_{C}-g_{C}) \end{array}$$

where (*e*_*C*_-*g*_*C*_) is the contribution of PID caused by other microorganisms. Note that to obtain the overall cumulative incidence for PID cases it is required that *x* > 0.

### Analysis

We compared the overall cumulative incidence of PID cases predicted by the model for each type of progression in intervention and control groups using the baseline values (Table 1). First, we examined the predicted cumulative incidences of chlamydial PID after one year when varying the fraction of chlamydia infection progressing to PID from 0 to 100%. Second, we used the maximum likelihood method to obtain the best fit estimate (and standard error) for the fraction progressing for each type of progression, using the observed cumulative incidences of PID cases in the trial. Third, we estimated the best fit (and standard error) for the fraction progressing to PID amongst women who were chlamydia positive at baseline, assuming that all PID cases were caused by *C. trachomatis*. The best fits for the models for the three types of progression were compared based on Akaike’s Information Criterion (AIC) (Additional file 1) [15]. Fourth, for each type of progression we used baseline values and the obtained maximum likelihood estimators to determine the time point since start of infection until half of the expected PID cases occurred (see Additional file 1 for more details).

**Table 1:** Parameter values describing the natural history of chlamydia infection, PID development and the screening intervention

**Parameters**	**Baseline values**	**Explanation**	**Sensitivity analysis**			**Source**
			**Distribution**	**Parameters**		
*Model parameters*						
*λ*		Force of infection (per day), calculated using^*^				
*1/r*	365	Mean duration of infection (days) [6,16]	N(μ,σ^2^)	μ=365	σ^2^=75^2^	Consensus
*p*	5.7%	Prevalence at baseline [11]	Bin(n,p)	n=2519	$\text{p}=\frac{143}{2519}$	[11]
*α*		Effective testing rate (per day), calculated using^†^				
*c*	22.2%	Coverage of testing uptake (per year) [11]	Bin(n,p)	n=2377	$\text{p}=\frac{527}{2377}$	[11]
*δ*	8.0%	Treatment failure [17]	U(a,b)	a=0%	b=50%	Consensus
*f*	estimated	Fraction of women becoming infected with chlamydia who will develop PID				
*Input parameter*						
*x*	30.0%	Proportion of PID cases due to chlamydia in control group [11]	Bin(n,p)	n=23	$\text{p}=\frac{7}{23}$	[11]

^*^In the absence of the tria (*α*=0), to observe chlamydia prevalence *p* at steady state: $\lambda =\frac{p}{1-p}r$.

^†^Reported uptake of chlamydia testing *c* during the follow-up period (outside of the trial) is reduced by the proportion with treatment failure *δ*, which results in the effective testing rate $\alpha =\frac{-ln\left(1-\left(1-\delta \right)c\right)}{365}$ per day [18].

N(μ,σ^2^), normal distribution (mean, variance); Bin(n,p), binomial distribution (size, probability); U(a,b), uniform distribution (minimum, maximum).

### Sensitivity analysis

A univariable sensitivity analysis was done for all model parameters and the proportion of PID cases caused by chlamydia. The parameters were varied within the 95% confidence interval using the distributions in Table 1. We obtained the maximum likelihood estimates for the fraction of women progressing to PID. Second, we did a multivariable sensitivity analysis by sampling each model parameter and the proportion of PID cases caused by chlamydia 1000 times from the distributions in Table 1. The maximum likelihood estimates for the fraction of women progressing to PID were determined and the quantiles (0.025 and 0.975) were obtained as 95% credibility intervals.

Third, we explored the effect of varying the mean time between start of infection and when progression to PID becomes possible. We do this in a model framework similar to the constant progression scenario. An additional parameter $\left(\tilde{f}\right)$ is needed to specify the fraction of women who develop PID at the time point when PID becomes possible. This differs from the fraction *f* in that $\tilde{f}$ refers only to the women who remain infected at the time point at which progression to PID becomes possible. We did not fit this model with the additional unknown parameter to the trial data as we have only two data points.We derived maximum likelihood estimates for the fraction $\left(\tilde{f}\right)$ for a fixed mean time between start of infection and progression to PID and report the corresponding fraction *f* (see Additional file 1 for more details).

Analytical results were derived in Mathematica and numerical solutions were obtained in R [19,20]. Code files can be obtained from the authors on request.

## Results

**Figure 2:**
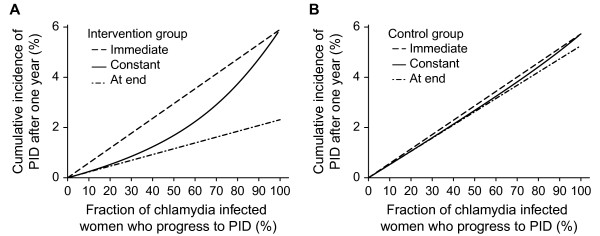
**Predicted cumulative incidence of chlamydial PID for the three types of timing of progression.** Panel **A**, results for intervention group; panel **B**, results for control group. Immediate progression (dashed line); constant progression (solid line); progression at the end (dashed-dotted line). The fraction progressing from chlamydia to PID is varied from 0-100% using baseline values for all other model parameters.

The predicted cumulative incidence of PID cases from chlamydia infection after one year depends on the fraction of chlamydia infected women who progress to PID and on the type of progression (Figure 2). In the intervention groups, the immediate progression scenario results in the highest cumulative incidence of PID, progression at the end the lowest, with intermediate values for the constant progression scenario (Figure 2A). In the control groups the predicted cumulative incidence of PID is similar for all three types of progression (Figure 2B).

In the immediate progression scenario, the predicted cumulative incidence of PID in the intervention and control groups is very similar; for any value of the fraction progressing to PID, women in both groups develop PID immediately after infection so testing and treating does not prevent any PID cases (Figure 2A, 2B). If the fraction progressing to PID is 100%, all women who become infected will progress to PID and the predicted cumulative incidence of PID after one year is similar to the baseline prevalence of chlamydia, because the mean duration of infection was assumed to be one year.

For scenarios of constant progression to PID or progression at the end of chlamydia infection, the predicted cumulative incidences of PID are similar if the fraction progressing to PID is low because the formulae describing PID incidence are similar when this value approaches zero (Figure 2A). For both of these scenarios, the incidence of PID depends on the number of infected women. The scenario with progression at the end always has a lower cumulative incidence than the other two, even when the fraction progressing to PID is 100%, because some of the infected women will have been effectively tested and treated before they clear the infection naturally, which is when they are at risk of developing PID.

Table 2 shows the maximum likelihood estimator (MLE) and the corresponding 95% CI for the estimated fraction of chlamydia infected women who progress to PID, using the observed cumulative incidences from the trial. The corresponding cumulative incidences of PID cases in the intervention and control groups shown are the best fitting values. For all types of progression to PID, the best fitting values for the fraction of women progressing to PID are between 8 and 10%. The AIC values are similar so the estimated fractions progressing to PID with all three types of progression are compatible with the data. Similar results were obtained considering only the point estimates of the cumulative incidence of PID cases of women who were chlamydia positive at baseline (results not shown).

**Table 2:** Estimated fraction progressing from chlamydia infection to PID, using baseline values

**Progression to PID**	Fraction progressing, % (95% CI)^*^	**Cumulative incidence of PID after one year, % (95% CI)**^**†**^		**Akaike’s Information Criterion**^**‡**^
		**Control group**	**Intervention group**	
*Data*				
Results from RCT		1.9 (1.2 to 2.9)	1.3 (0.7 to 2.1)	
*Model*				
Immediate progression	8.3 (5.7 to 11.0)	1.6 (1.1 to 2.1)	1.6 (1.1 to 2.1)	13.3
Constant progression	9.9 (6.8 to 13.0)	1.7 (1.2 to 2.3)	1.5 (1.0 to 1.9)	12.1
Progression at the end	10.0 (6.8 to 13.1)	1.7 (1.2 to 2.3)	1.5 (1.0 to 1.9)	12.1

^*^The 95% CI is obtained by using the corresponding standard error and assuming a normal distribution.

^**†**^Cumulative incidence of PID after one year caused by chlamydia and other microorganisms together.

^‡^ Akaike’s information criterion values describe fit of model.

PID, pelvic inflammatory disease; RCT, randomised controlled trial; CI, confidence interval.

In the scenario of constant progression to PID, with a constant daily risk of developing PID, it takes 228 days until half of the expected PID cases are observed and for the progression at the end it takes 253 days, using the MLE in Table 2 (see Additional file 1 Figure A1). In the immediate progression scenario, it takes 0 days which is an intuitive consequence of progression without a delay.

### Sensitivity analysis

**Figure 3:**
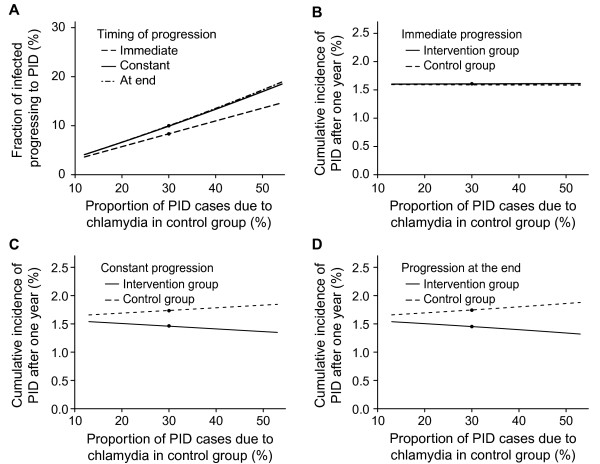
**Univariable sensitivity analysis, varying the proportion of PID cases due chlamydia in the control group.** Panel **A**, fraction of progression needed in each type of timing of progression: immediate progression (dashed line); constant progression (solid line); and progression at the end (dashed-dotted line). Panels **B**- **D**, cumulative incidences of PID cases caused by chlamydia and other microorganisms after one year in the control group (dashed line) and in the intervention group (solid line) for the three types of timing of progression: immediate progression (B); constant progression (C); and progression at the end (D). The baseline value scenario is indicated with a black dot. Proportion of PID cases due chlamydia infection in the control group from 13-53% using baseline values for all other parameters. The observed cumulative incidences of PID after one year (%) in the trial were: control group 1.9 (95% CI 1.2 to 2.9), intervention group 1.3 (95% CI 0.7 to 2.1).

In the univariable analysis the proportion of PID cases due to chlamydia in the control group is the most influential parameter affecting the best fit for the fraction progressing to PID (Figure 3). Within the range of values sampled, the fraction progressing to PID varies from 4 to 19% (Figure 3A). The cumulative incidences of PID cases caused by chlamydia and other microorganisms after one year (Figure 3B-D) are also influenced for the scenarios of constant progression or progression at the end of infection but only marginally for immediate progression. Varying the duration of chlamydia infection or the baseline prevalence influences the force of infection but results in relatively small changes in the fraction progressing or the cumulative incidence of all-cause PID. Changing the percentage with treatment failure or the uptake of testing during follow up has marginal influence (results not shown). In the multivariable sensitivity analysis the means over all parameter sets for the fraction progressing to PID were similar to those in the baseline analysis (see Additional file 1 Figure A2). In the additional model framework the corresponding best fitting values for the fraction of infected women developing PID (*f*) were in the same range as the main three types of progression (see Additional file 1 Figure A3).

## Discussion and conclusion

This study used a mathematical model to simulate the results of a randomised controlled trial of a chlamydia screening intervention. The predicted cumulative incidence of PID was lower in the intervention than the control group if progression to PID occurred at a constant rate or at the end of chlamydia infection. If progression to PID occurs immediately after chlamydia infection, screening and treatment do not reduce the cumulative incidence of PID. The model estimates, for constant progression and progression at the end, that 10% (95% CI 7-13%) of chlamydia infections progress to PID.

A strength of this study was the use of a dynamic mathematical model to investigate the timing of progression from chlamydia infection to PID. There were, however, several simplifying assumptions. First, it is not biologically plausible for chlamydia to ascend in the genital tract either immediately after endocervical infection or just before natural clearance. These extreme situations were chosen to represent progression early and late in the course of chlamydia infection. Other plausible possibilities about the timing of progression, e.g. assuming a woman has to be infected for a certain time period before being at a constant daily risk of developing PID, were not investigated because we did not have enough data to fit models with more than one unknown parameter. Second, we counted the number of PID episodes rather than the number of women developing PID. The model structure assumed that PID could happen repeatedly in the same woman but that a history of PID did not influence the course of chlamydia infection, susceptibility to chlamydia or future progression to PID. These assumptions might not be true but, since both the trial follow-up period and baseline value for the mean duration of chlamydia infection were one year, there was a negligibly small percentage of women with repeated chlamydia infections or PID episodes in the model. Third, it was assumed that antibiotic treatment was specific to *C. trachomatis*, which is not the case. Azithromycin is also active against *Mycoplasma genitalium* but a causal association with PID is still debated so it was not possible to estimate the potential effect of treatment on other microorganisms [2,21]. Finally, we considered a closed population; this was a reasonable assumption because very few women in the trial were lost to follow-up.

The use of empirical data from a randomised controlled trial was also an advantage. The Prevention of Pelvic Infection study is the only trial with data about the baseline prevalence of chlamydia in the control group, which allowed us to investigate the incidence of PID amongst untreated women. There are also limitations to the trial. Although discussed previously [11], we restate limitations as they apply to our study here. First, the point estimates of PID incidence were rather imprecise, owing to the lower than expected incidence of PID in the trial [11]. The relative reduction in PID incidence in the Prevention of Pelvic Infection study was consistent with, but smaller than in the other two randomised trials [9,10], probably because of a lower risk of methodological bias; another possibility is the high testing uptake during the follow-up period in both groups [3,11]. When using the maximum likelihood method to estimate the fraction progressing to PID, the best fit values for the cumulative PID incidence rates in the control and intervention groups were closer than observed in data. The value for the control group was, nevertheless, higher than for the intervention group for the model assuming a constant rate of progression. Second, we only used the values for the 12-month incidence of PID to fit the model, rather than individual dates of PID diagnosis. These dates were collected retrospectively, by self-report backed by medical records, but were limited to the date when the participant presented to a healthcare facility and was diagnosed with PID, and were not accurate enough to construct a survival curve. Third, only symptomatic PID cases were observed so the cumulative incidence of PID cases could have been underestimated. This would lead to an underestimation of the fraction of women becoming infected with chlamydia who will develop PID.

There are very few mathematical modelling studies that consider explicitly how the timing of progression to PID might affect the outcome of chlamydia screening interventions [13]. Smith and colleagues examined different intervals for the development of PID following a chlamydia infection using a Markov model [22]. They used data from a prospective cohort study of women at high risk of PID [23,24]. Our study addresses the suggestion of Smith et al. to investigate PID development time in women at low risk of chlamydia comparing data about PID rates from different screening strategies. Our findings also support those of Smith et al., with the most cases of PID averted with the longest development time. Our study estimated that 8-10% of women with chlamydia infection develop PID, which corresponds to the estimate of Adams and colleagues, based on data about clinical PID reports from primary care [25], but lower than the estimated progression fraction assumed in many cost-effectiveness studies [13]. The baseline value of 30% (7/23) for the proportion of PID cases due to chlamydia infection in the trial is in line with what has been reported in the literature [2,3].

A constant rate of progression from chlamydia to clinically diagnosed PID or progression at the end of the course of chlamydia was compatible with the findings of the Prevention of Pelvic Infection trial. The two scenarios differ conceptually, however, regarding the window of opportunity for screening to prevent PID. In the scenario with progression at the end of infection, the time window for preventing PID is the whole infection period. In the constant progression scenario, the time window might be shorter than the duration of infection. The constant rate assumes that the time between start of infection and developing PID follows an exponential distribution. This implies that some women will develop PID soon after infection whereas others will develop it very late in their infection. In practice, there would always be some unpreventable chlamydial PID as the screening interval cannot be made short enough to find each infected woman before she progresses. Progression at the end of the course of chlamydia infection is probably less biologically plausible than constant progression. Progression early in the course of chlamydia infection, represented in the model as immediate progression, was the least likely. This differs from the findings from animal models in which progression in the mouse model happens by 24 hours [12] and in the guinea pig model within the first week [26]. It is possible that *C. trachomatis* ascends early in the course of infection in humans but that clinical PID is observed later. However, if most chlamydia infections in women progressed so early in the course of infection, many clinical PID cases would be expected to have occurred before detection of prevalent infections through screening [27]. The development of PID symptoms and clinical diagnosis have to be able to happen over a longer time course for screening to achieve reductions in the incidence of PID of 35% [11] or more [9,10], given that only 30% of PID cases are caused by chlamydia and that PID resulting from a new infection during the follow-up period cannot be prevented [28]. Most women with PID in the trial reported sexual intercourse with two or more partners during the year. Since bacterial vaginosis is thought to promote ascending *C. trachomatis* infection [23], it could be hypothesised that sex with a new partner alters the composition of vaginal flora and encourages progression of prevalent endocervical chlamydia to PID.

This study has implications for future research and practice. The relatively low estimated fraction of asymptomatic chlamydia progressing to clinical PID can be used to give advice to women with chlamydia infection. The uptake of the screening interventions in randomised controlled trials was much higher than uptake rates observed in practice [29,30]. We plan to conduct future modelling studies that investigate the impact of achievable levels of chlamydia screening on the interruption of ascending chlamydia infections using a model that can also examine the effect of differences in the timing of progression. The numbers of PID cases prevented could then be compared to those prevented indirectly as the result of reduced exposure to chlamydia. The findings of this study suggest that clinical PID can occur throughout the course of a chlamydia infection, which leaves a window of opportunity for screening to prevent PID.

## Abbreviations

PID: Pelvic inflammatory disease; chlamydia: Chlamydia trachomatis infection; POPI: Prevention Of Pelvic Infection; CI: Confidence interval; AIC: Akaike’s Information Criterion.

## Competing interests

The author(s) declare that they have no competing interests.

## Authors' contributions

SAH, CLA, JCMH, and NL developed the idea for the study and analyzed the model. PO, SK, and PH provided detailed data and insights to the RCT. SAH programmed the model and wrote the first draft of the paper. All authors commented on the manuscript and approved the final version.

## Pre-publication history

The pre-publication history for this paper can be accessed here:

http://www.biomedcentral.com/1471-2334/12/187/prepub

## Supplementary Material

Additional file 1 Appendix. Appendix for method and result section. Click here for file
