# Supplementary material for: Timing of progression from Chlamydia trachomatis infection to pelvic inflammatory disease: a mathematical modelling study
Source: BMC Infect Dis. 2012 Aug 11;12:187. doi: 10.1186/1471-2334-12-187 (PMC3505463; doi:10.1186/1471-2334-12-187)
Supplement: Additional file 1 — Appendix. Appendix for method and result section. [file 1471-2334-12-187-S1.pdf]

Timing of Progression of *Chlamydia trachomatis* Infection to Pelvic Inflammatory Disease: A Mathematical Modelling Study

Sereina A. Herzog, Christian L. Althaus, Janneke C.M. Heijne, Pippa Oakeshott, Sally Kerry, Phillip Hay, Nicola Low

Additional File 1

Content

1. Initial conditions and cumulative incidence of pelvic inflammatory disease (PID).. 2
2. Maximum likelihood estimation of the fraction f of chlamydia infections progressing to PID (f_{MLE}) 3
3. Akaike's information criterion (AIC).. 3
4. How incidence of PID accumulates in each type of progression 3
5. Result of multivariable sensitivity analysis... 5
6. Varying mean time between start of infection and time point when progression to PID becomes possible........ 6
7. Appendix references.. 7

1. Initial conditions and cumulative incidence of pelvic inflammatory disease (PID)

The function for the cumulative incidence of PID cases $C(t)$ at time point t depends for each type of progression (immediate progression, constant progression, progression at the end) on the specific incidence of PID cases and fulfils that $C(0)=0$.

$$C_{immediate}(t) = f\lambda \int_0^t S(\tau)d\tau$$
$$= \frac{f\lambda}{\alpha + r + \lambda}\left[(\alpha + r)(s_1 + s_2)t + \frac{e^{-(\alpha+r+\lambda)t}((\alpha + r)s_2 - \lambda s_1)}{\alpha + r + \lambda}\right] - \frac{f\lambda((\alpha + r)s_2 - \lambda s_1)}{(\alpha + r + \lambda)^2}$$

$$C_{constant}(t) = \gamma \int_0^t I_1(\tau)d\tau$$
$$= \gamma\left[\frac{\lambda(r + \alpha)(s_1 + s_2 + s_3)}{(\alpha + r + \gamma)(\alpha + r + \lambda)}t - \frac{e^{-(\alpha+r+\lambda)t}\lambda\big((\alpha + r)(s_2 + s_3) - \lambda s_1\big)}{(\alpha + r + \lambda)^2(\lambda - \gamma)} + \frac{e^{-(\alpha+r+\gamma)t}(s_2(\alpha + r + \gamma)\gamma + s_3(r + \alpha)\lambda - \gamma\lambda(s_1 + s_2))}{(\alpha + r + \gamma)^2(\lambda - \gamma)}\right]$$
$$- \frac{\gamma}{\lambda - \gamma}\left[\frac{(s_2(\alpha + r + \gamma)\gamma + s_3(r + \alpha)\lambda - \gamma\lambda(s_1 + s_2))}{(\alpha + r + \gamma)^2} - \frac{\lambda\big((\alpha + r)(s_2 + s_3) - \lambda s_1\big)}{(\alpha + r + \lambda)^2}\right]$$

$$C_{end}(t) = fr \int_0^t I(\tau)d\tau$$
$$= \frac{fr}{\alpha + r + \lambda}\left[\lambda(s_1 + s_2)t + \frac{e^{-(\alpha+r+\lambda)t}\big((\alpha + r)s_2 - \lambda s_1\big)}{\alpha + r + \lambda}\right] - \frac{fr\big((\alpha + r)s_2 - \lambda s_1\big)}{(\alpha + r + \lambda)^2}$$

where s_1=S(0), s_2=I_1(0) and s_3=I_2(0) describe the initial conditions for the constant progression scenario. The intervention group was treated for chlamydia infections and therefore has $s_1 = 1 - p * \delta$, $s_2 = p * \delta \frac{r}{r+\gamma}$, $s_3 = p * \delta \frac{\gamma}{r+\gamma}$. The control group starts at steady state in the absence of the trial (α=0) with $s_1 = 1 - p$, $s_2 = p \frac{r}{r+\gamma}$ and $s_3 = p \frac{\gamma}{r+\gamma}$. The initial conditions for immediate progression and progression at the end of infection (where $\gamma = 0$ and $I(t) = I_1(t) + I_2(t)$) are described as S(0)= s_1 and $I(0) = s_2 + s_3$.

2. Maximum likelihood estimation of the fraction *f* of chlamydia infections progressing to PID (f_{MLE})

The maximum likelihood estimate [1] of the fraction *f* of infected women who progress from chlamydia to PID is obtained by maximising the following likelihood *L*. We used subscript *I* in the likelihood to indicate the intervention group and subscript *C* for the control group. We assumed for both groups that the observed PID cases (o_I, o_C) are binomially distributed and that they are a mixture of cases caused by chlamydia and by other microorganisms. The probability for each woman to develop PID within the timeframe of one year is set to equal the overall cumulative incidence of PID cases after one year (e_I, e_C).

$$L = \prod_{j\in\{I,C\}} \binom{N_j}{o_j} {e_j}^{o_j} (1 - e_j)^{N_j - o_j}$$

where N_j equals the number of women in each group. The derivations of e_I and e_C are explained in the method section of the main text.

3. Akaike's information criterion (AIC)

The Akaike's information criterion (AIC) is defined as

$$AIC = -2\log(L) + 2k$$

where *L* is the likelihood and *k* the number of estimated parameters in the model, k=1 for the model used in this study [2].

4. How incidence of PID accumulates in each type of progression

To illustrate how incidence of PID accumulates from the start of infection, a hypothetical cohort of women who became infected at the same time is followed over time. In this hypothetical cohort, women can develop PID according to the three processes described in the main text. Figure A1 shows the cumulative incidence of PID as a function of time since infection, using baseline values (see Table 1 in main text) and the maximum likelihood estimates (MLE) for the fraction progressing to PID (f_{MLE}). We obtained the time point where half of the expected PID cases ($f_{MLE}/2$) have occurred (T_{50} in Table A1) and indicated them with black dots in Figure A1.

Table A1 Time until half of expected PID cases occured

Type of progression	f_{MLE} *	T_{50} †
Immediate progression	8.3 %	0 days
Constant progression	9.9 %	228 days
Progression at the end	10.0 %	253 days

* MLE for fraction progressing to PID from Table 2.
† Time point when half of the expected PID cases ($f_{MLE}/2$) have occurred.

PID, pelvic inflammatory disease; MLE, maximum likelihood estimate.

In the immediate progression scenario, the time point when half of the expected PID cases occurred is 0 days. This is an intuitive consequence of the progression without a delay.

In the scenario with a constant daily risk of developing PID (constant progression) the cumulative incidence of PID is increasing fast in the beginning and flattens out (Figure A1). It takes 228 days until half of the expected PID cases are observed. In the progression at the end scenario, a similar behaviour for the cumulative incidence can be observed (Figure A1) and the half-value time (T_{50}) is simply the half-life of infectious duration, i.e. 253 days (=ln(2)/r).

These values look similar because the duration until PID development is exponentially distributed in both scenarios but the definition of the duration until PID development becomes possible differs. In the progression at the end scenario, the duration until PID development equals the duration of the infection as PID develops just before natural clearance. In the constant progression, PID can develop throughout the infection which implies that some women will develop PID soon after infection whereas others will develop it very late in their infection.

Despite the similarities in the results about T_{50}, the two scenarios differ conceptually regarding the window of opportunity for screening to prevent PID. In the progression at the end scenario the time window for preventing PID is the whole infection period whereas in the constant progression scenario the window might be shorter than the duration of infection.

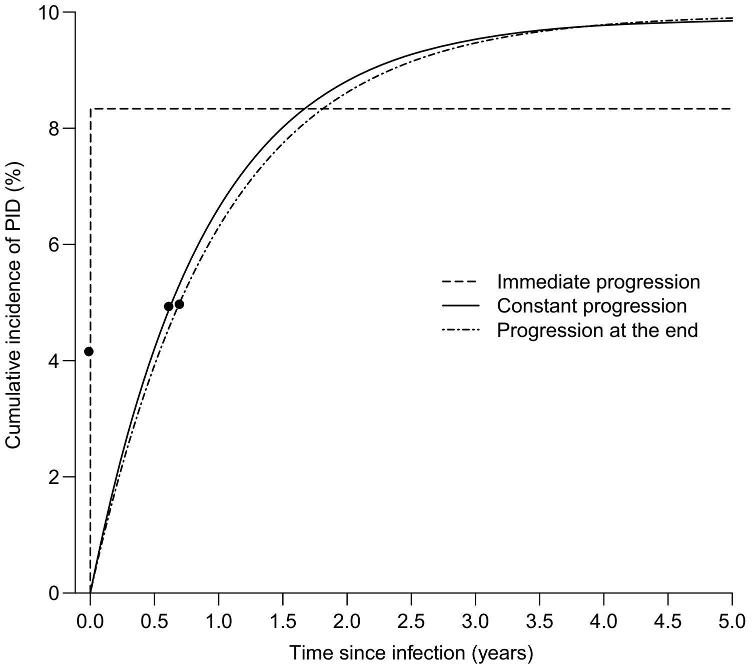

Figure A1 Illustration how incidence of PID accumulates in each type of progression. Cumulative incidence of PID following a hypothetical cohort of women who become infected at the same time, using baseline values and the maximum likelihood estimates for fraction progressing (f_{MLE}): immediate progression (dashed line); constant progression (solid line); and progression at the end (dashed-dotted line). The black dots indicate when half of the expected PID cases occurred ($f_{MLE}/2$).

5. Result of multivariable sensitivity analysis

We did a multivariable sensitivity analysis by sampling each model parameter and the proportion of PID cases caused by chlamydia 1000 times from the distributions in Table 1 in the main text. The maximum likelihood estimates for the fraction of women progressing to PID (*f*) were determined and the quantiles (0.025 and 0.975) were obtained as 95% credibility interval, see Figure A2.

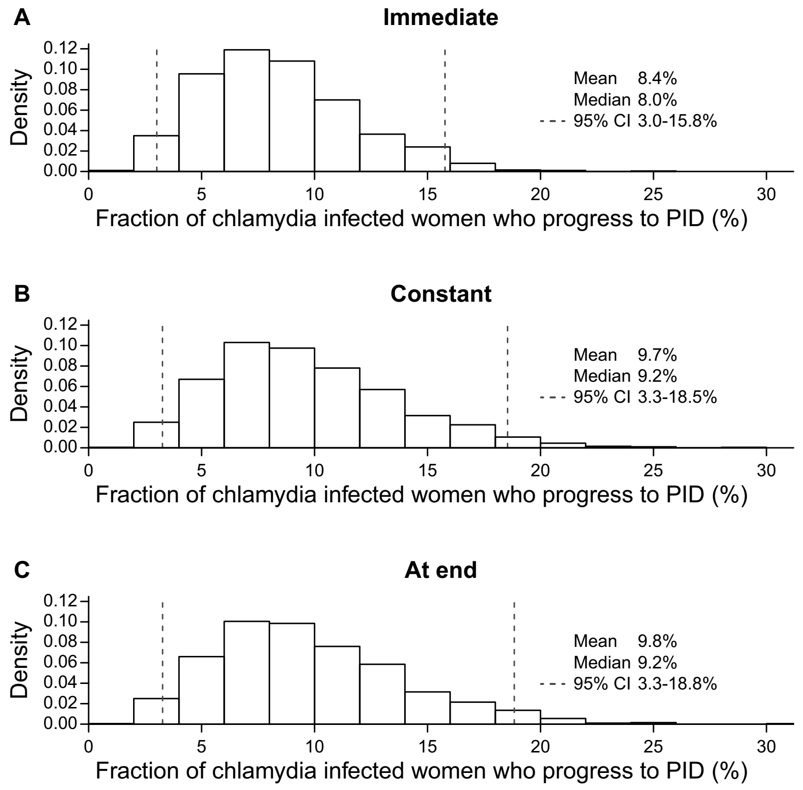

Figure A2 The maximum likelihood estimations for the fraction *f* of progression for the sensitivity analysis with 1000 parameter sets: immediate progression (A); constant progression (B); and progression at the end (C). The mean and the median of the maximum likelihood estimations are listed within each panel and the 95% credibility interval (CI) is shown (dashed line).

6. Varying mean time between start of infection and time point when progression to PID becomes possible

For the sensitivity analysis we introduced a model framework which is similar to the constant progression scenario but allows varying the mean time between start of infection and the time point when progression to PID becomes possible. We used the same model structure as described in the main text with two stages of infections (I_1, I_2) but in doing so the interpretation of I_2 has changed.

In stage I_1 women are infected without having PID but those who make the transition to stage I_2 with rate γ are now at risk to develop PID, i.e. upon entering I_2 a fraction $\tilde{f}$ will develop PID. Stage I_2 is therefore a mixture of infected women with PID ($\tilde{f}$) and without PID (1-$\tilde{f}$) and consequently incidence of chlamydial PID equals $\tilde{f}\gamma I_1$.

The introduction of the new parameter $\tilde{f}$, fraction progressing to PID at transition to I_2, is needed to specify the fraction of women who develop PID at the time point when PID becomes possible. This differs to the fraction f in that $\tilde{f}$ refers only to the women who remain infected at the time point at which progression to PID becomes possible. The interpretation of the fraction f is the same as in the three types of progression of the main text, i.e. f is the fraction of all women infected that will develop PID in the absence of an intervention The relationship between f and $\tilde{f}$ is

$$f = \tilde{f}\frac{\gamma}{r+\gamma}$$

where $\frac{\gamma}{r+\gamma}$ is the probability to make the transition from I_1 to I_2, r is the clearence rate of the infection, and $1/\gamma$ is the mean time between start of infection and the time point when progression to PID becomes possible. The relationship implies for a short mean duration until progression to PID becomes possible (i.e. small $1/\gamma$) that $\tilde{f}$ will be similar to f. But for increasing $1/\gamma$ follows that $\tilde{f}$ has to increase too as women will have recovered without making the transition to I_2. Note, there is a maximum value for the mean time $1/\gamma$ because the conditions $\tilde{f} \leq 100\%$ and $f \leq 100\%$ have to be fulfilled.

The scenario of immediate progression can be approximated with $1/\gamma \approx 0$ which implies that $\tilde{f} \approx f$; and the constant progression scenario corresponds to the situation with $\tilde{f}$=100%. The scenario with progression at the end of the infection cannot be approximated in this model framework.

We could not fit both unknown parameters (γ, $\tilde{f}$) to the trial data as we have only two data points. We derived the maximum likelihood estimate (MLE) for the fraction of infected women developing PID at the transition to I_2 ($\tilde{f}$) for different values for the mean duration ($1/\gamma$), using the observed cumulative incidences from the trial and the baseline values for all other paramters (see Table 1 in the main text). We varied the mean duration ($1/\gamma$) between 0 years and a maximum of 9.1 years, which is a consequence of the condition $\tilde{f} \leq 100\%$. For each mean duration value we obtained MLE of fraction $\tilde{f}$, the corresponding MLE of fraction f (using the relationship stated above), and the AIC value. We also obtained the cumulative incidence of PID after one year for the intervention group and the control group.

The best fitting values for the fraction of all infected women developing PID (f_{MLE}) are between 8.3% and 9.9% for varying the mean time between start of infection and the time point when progression to PID becomes possible (dashed line in panel A of Figure A3). Note, the estimates are in the same range as the three types of progression in the main text. The corresponding cumulative incidence of PID after one year are very similar for the intervention and control group if the mean time between start of infection and progression is short but diverge with increasing mean duration

(panel B). The AIC values are similar for the explored ranges of the mean duration (note the narrow scale of the y-axis in panel C), But the best fit model converges to the scenario when PID develops at a constant rate throughout the infection, i.e. $\tilde{f} = 100\%$.

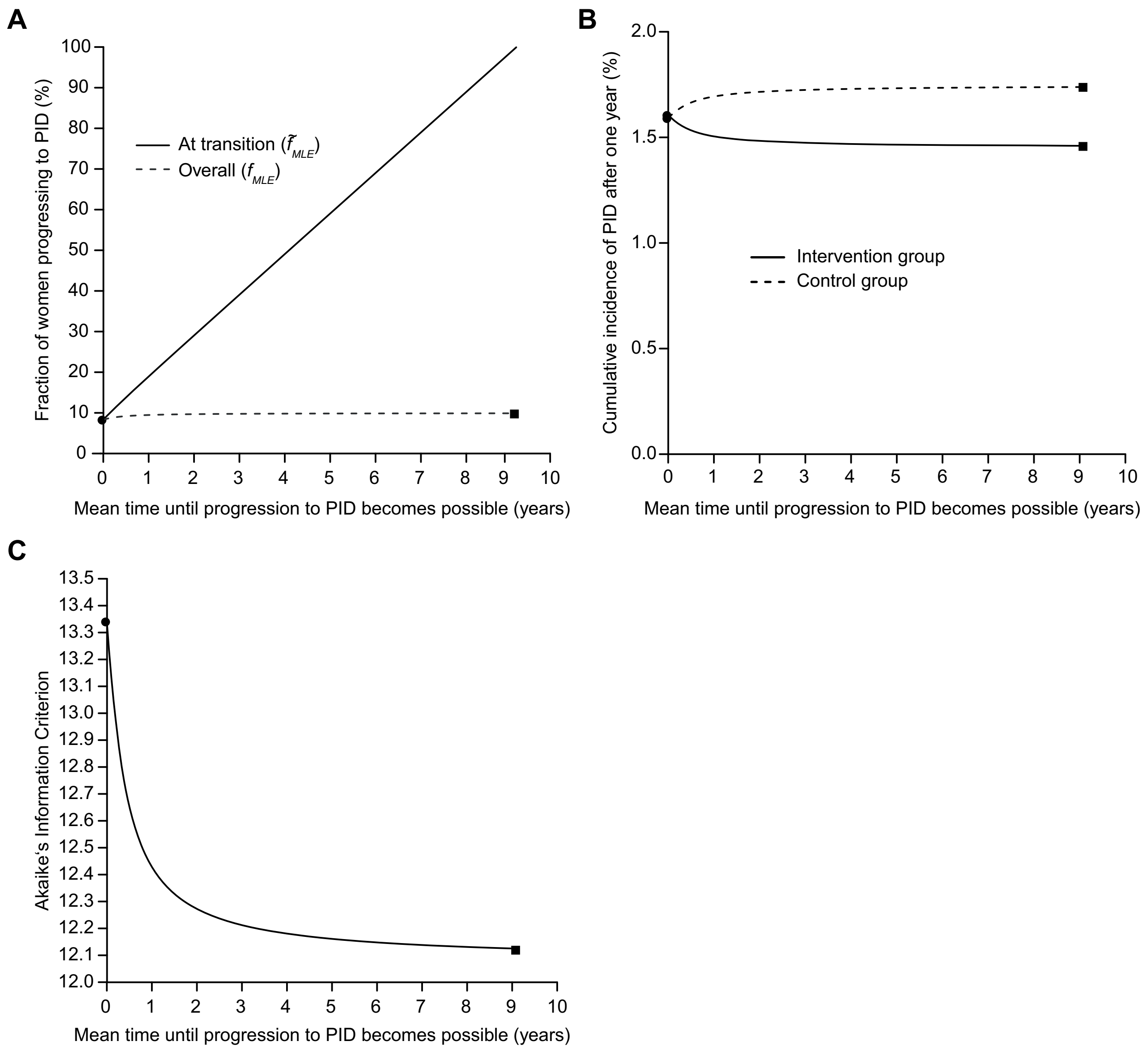

Figure A3 Varying mean time between start of infection and time point when progression to PID becomes possible ($1/\gamma$), using baseline values. Panel A, maximum likelihood estimators (MLE) for $\tilde{f}_{MLE}$ respectively f_{MLE}; panel B, corresponding cumulative incidence of PID after one year for the MLE values for intervention group (solid line) and control group (dashed line); panel C, corresponding Akaike's Information Criterion (AIC) values for the MLE values. In all panels, the black dot indicates the approximation of the immediate progression and the black quadrate corresponds to the scenario of constant progression.

7. Appendix references

1. Kirkwood BR, Sterne JAC: *Essential medical statistics*. Malden, Mass: Blackwell Science; 2003.
2. Burnham KP, Anderson DR: *Model selection and multi-model inference: A practical information-theoretic approach*. New York, NY: Springer; 2010.
